# Microbial communities on dry natural rocks are richer and less stressed than those on man-made playgrounds

**DOI:** 10.1128/spectrum.01930-24

**Published:** 2025-04-09

**Authors:** J. Manninen, M. Saarenpää, M. Roslund, P. Galitskaya, A. Sinkkonen

**Affiliations:** 1 Faculty of Biological and Environmental Sciences, Ecosystems and Environment Research programme, University of Helsinki 89214 , Helsinki, Finland; 2 Natural Resources Institute Finland Luke 419837, Helsinki, Finland; 3 Research Institute for Environmental Studies 667768 , Parede, Portugal; University of Minnesota Twin Cities, St. Paul, Minnesota, USA

**Keywords:** biodiversity hypothesis, urban environment, microbial community composition, soil microbiota, urban microbiome, planetary health, microbial exposure, play environments

## Abstract

In modern urbanized societies, the incidence of major immune-mediated diseases is several times higher than before World War II. A potential explanation is that these diseases are triggered by limited possibilities to be exposed to rich environmental microbiota. This requires that the urban environment hosts less and poorer microbiota than the natural environment. The current study was designed to test the assumption that urban man-made environments host less and poorer environmental microbiota, compared to natural habitats. We selected two types of dry environments, natural rocks and playground rubber mats, both of which were used daily and extensively by children. In quantitative PCR and next-generation sequencing, bacterial abundance and richness were higher on the natural rocks than the rubber mats. Altogether, 67 amplicon sequence variants (ASVs) belonging mostly to Actinobacteria and Proteobacteria were indicative of rock microbiota, while three ASVs were indicative of rubber mats. Interestingly, bacteria formed more complex networks on rubber mats than natural rocks. Based on the literature, this indicates that the studied artificial dry environment is more challenging and stressful for bacterial communities than dry natural rocks. The results support the hypothesis that urban man-made environments host poor microbial communities, which is in accordance with the biodiversity hypothesis of immune-mediated diseases.

**IMPORTANCE:**

The current study provides new evidence that artificial urban play environments host poor microbial communities and provide a stressful environment for microbes, as compared to dry natural rocks. Through this, the current study underlines the need to enhance microbial diversity in urban areas, especially in outdoor play environments, which have a crucial role in providing essential microbial exposure for the development of children’s immune system. This research can potentially offer guidance for urban planning and public health strategies that support planetary health.

## INTRODUCTION

As a result of urbanization, natural vegetated living environments have been replaced by gray zones characterized by buildings and sealed surfaces (1). The most dramatic distortion of natural ecosystems is the replacement of natural soil and vegetation with artificial man-made surfaces, like concrete, asphalt, and rubber (2). According to the European Environment Agency (3), almost half of the area in cities in the European Union is covered by man-made, non-permeable surfaces. These materials typically dry quickly, and diurnal moisture and temperature variation is exaggerated compared to natural soil surface.

Within the European Union, playground rubber mats are one of the rare materials that fill the safety requirements for shock absorption of safety platforms (SFS-EN 1177:2018). Surprisingly, the microbial communities of rubber mats have never been compared to those on natural soils. In the context of planetary health, it may not be optimal to compare playground rubber mats with moist or nutrient-rich habitats that are known to contain diverse microbiota, like natural forest soils, but merely with natural dry habitats, particularly rocks. Rocks have extreme moisture and temperature variation compared to biofilm-rich habitats, like organic soil.

The replacement of natural surface soil with man-made materials plausibly affects urban microbial ecosystems. In our earlier intervention trials, the introduction of microbially rich organic soil and diverse vegetation to urban daycare yards changed microbial communities to resemble those in natural forests (4–6). In recent decades, several hypotheses have connected the increasing incidence to decreased exposure to microbes in the urban environment (7, 8). According to the biodiversity, hygiene, and Old Friends’ hypotheses (9–12), commensal microbiota is altered, and the maturation of the immune system is often distorted when the exposure to rich environmental microbiota commonly found in vegetated areas is limited, particularly during early years of life (4, 13–18). In the planetary health context, it is thus essential to understand how soil sealing affects urban environmental microbial communities.

The biodiversity hypothesis of immune-mediated diseases can be divided into several assumptions that can be studied one by one. In addition to assumptions related to commensal microbiota, immune response, and disease incidence, which have been tested or are being tested by our group (4–6, 19–22), the biodiversity hypothesis is built on the assumption that the possibilities for contacts with environmental microbial diversity are poorer among urban than rural children. Surprisingly, this assumption has not been extensively tested. There are no published studies that compare environmental microbial communities in man-made and natural outdoor play environments. It is known that the transfer of environmental microbiota indoors is weak among urban seniors compared to their rural counterparts (23, 24), that airborne green exposure enhances microbial community of the skin and respiratory tract (25, 26), and that just 45 min of exposure to green areas next to schoolyards repaired and enriched the children’s disturbed microbiome faster than being near school’s sports fields (27). Although those findings will be important for future urban planning and management, they consider urbanites or indoor environments. In addition to such tests, we need knowledge on whether environmental microbiota is poor on man-made surfaces. Therefore, microbial community composition and abundance on these surfaces should be studied.

In this study, we compared dry environments, urban natural rocks, and artificial rubber mats. The rubber mats were daycare or schoolyard playgrounds, and natural rocks were either next to them or otherwise well-known free time destinations preferred by children in the areas of Lahti and Helsinki, Finland. We examined whether the diversity of the microbiome of dry habitats in urban environments is different in natural material compared to artificial material. Our hypotheses for the present study were that the richness of the bacterial communities and the abundance of health-beneficial taxa were higher on natural rocks than on playground rubber mats. We also assumed that bacterial genera form different co-occurrence networks depending on whether the habitat is natural or artificial. The ultimate goal was to study the environmental microbial exposome in urban areas and test one of the key assumptions of the biodiversity hypothesis of immune-mediated diseases: do urban man-made surfaces comprise poorer microbial communities than natural habitats with similar weather and moisture variation?

## MATERIALS AND METHODS

### Experimental setup and sampling

Surface soil (0 mm–3 mm) was collected, altogether 28 dust and dirt samples, obtained from 19 playground rubber mats and 9 natural rocks situated in built environments (samples A1–A19 and N1–N9, A = artificial rubber mat soil, N = natural rock soil). Among 28 samples, 14 represented seven pairs (A1–A7 and N1–N7) where dust and dirt were taken from the same sampling area (Table S1). These areas were local schools or playgrounds that had a natural rock within 100 m from yards. The materials of the rubber mats were styrene-butadiene rubber and ethylene propylene diene monomer rubber. The sampled rocks were either biotite paragneiss, microcline granite, or quartzite (28). All the natural rocks and playground rubber mats are known to be in frequent use by children. The samples were collected in July 2021 from two Finnish cities, Lahti and Helsinki. The weather was sunny and partly cloudy, and the daytime temperature was above 20°C. July 2021 was warmer than usual, and the mean temperature was 1°C–4°C higher than average. It was also dry, with precipitation falling between 30 and 80% of the long-term average. This was also preceded by low precipitation and dryness in June (Finnish Meteorological Institute: http://www.ilmastokatsaus.fi).

Two dust samples were collected into separate zip lock bags from each playground rubber mat and natural rock, consisting of three sub-samples within 50 cm–100 cm from each other. Sterile polyethylene toothbrushes and a sterilized tablespoon were used for sampling. For rubber mats, one sample was taken from the most central part of the rubber mat, such as in front of the football goals, next to the climbing frame, or under the slide (Table S1). Another sample was taken from the edge of the rubber mat, near the entry point. From the rocks, one sample was collected from the top of the rock and another sample from the rock plateau. The samples were put in a cool bag with ice packs, frozen on the same day at –20°C, and stored at −80°C within 2 days until further processing. DNA was extracted and amplified in 2 months from collection and sent to sequencing right after.

### Quantitative PCR (qPCR)

Quantitative PCR of the bacterial 16S rRNA gene was performed using SYBR Green I binding and conducted on a Light Cycler 96 Quantitative real-time PCR machine (MJ Research, MA, USA). The forward primer, pE: 5´-AAA CTC AAA GGA ATT GAC GG-3, and the reverse primer, pF: 5′-ACG AGC TGA CGA CAG CCA TG-3 (Metabion), were utilized. Each sample was amplified in triplicate in 20 µL reactions consisting of 10 µL 2× Powertrack SYBR green mastermix (Thermo Scientific, MA, USA), 0.2 µL 20 mg/mL bovine serum albumin (BSA), 0.5 µL of each primer (10 µmol/L), and 2.0 µL of the sample template.

To enable quantification of the number of bacterial 16S rRNA gene copies in the original sample, a standard curve was included in every run. The qPCR cycling protocol was as follows: an initial denaturation step at 95°C for 2 min, followed by 33 cycles of denaturation at 95°C for 10 s, annealing at 50°C for 20 s, and extension at 72°C for 30 s. Melting curve analysis of the amplicon was conducted with the following parameters: 95°C for 10 s, 65°C for 60 s, 97°C for 1 s, and 37°C for 30 s, while continuously measuring the fluorescence signal. For the positive control, a mock (ZymoBIOMICS Microbial community DNA standard), 200 ng in a 20 µL volume, cat, no. D6305 (Zymoresearch), was used. The stock solution was diluted 1:100 in sterile water. Sterile water was used as the negative control.

### Sample preparation for MiSeq sequencing

Bacterial communities in the samples were analyzed using Illumina MiSeq 16S rRNA gene metabarcoding with read length 2 × 300 bp using a v3 reagent kit. Soil samples for MiSeq sequencing were prepared as in Parajuli et al. (24). Samples were extracted with the PowerSoil DNA Isolation Kit (Qiagen, Hilden, Germany) according to the manufacturer’s standard protocol. The quality of the extracted DNA was checked using agarose gel (1.5%) electrophoresis and quantified with Quant-iT PicoGreen dsDNA reagent kit (Thermo Scientific, MA, USA). The DNA concentration was adjusted to 0.4 ng/µL for each sample. The V4 region within the 16S rRNA gene was amplified in PCR (three replicates from each sample) using 515F and 806R primers (29). Negative controls were included at all steps (sampling, DNA extraction, PCR, and sequencing controls) and positive controls at each PCR. Sterile water was used as the negative control. The positive control was *Cupriavidus necator* JMP134, DSM 4058. The sampling controls were also tested to ensure that they did not contain DNA. The PCR products were purified using Agencourt AMPure XP solution (Beckman) (30) and then targeted in the secondary PCR (TagPCR).

### Sequence processing

Sequencing data from surface soil samples were processed and analyzed using Mothur (version 1.48.0) (31). The sequence processing protocol mainly followed the MiSeq standard operating procedure suggested by Kozich et al. (32). Sequences were trimmed and screened to remove any mismatches to primers or DNA-tag sequences, ambiguous bases, and homopolymers longer than 8 bp. Bacterial sequences were aligned against a SILVA reference (version 132 [33]). Preclustering minimized sequencing errors (34) and grouped sequences into amplicon sequence variants (ASVs). Chimeras were removed using the VSEARCH algorithm (35). Sequences classified to Chloroplast, Mitochondria, unknown, Archaea, and Eukaryota were removed from the analyses. Low abundance ASVs (≤10) were removed from the sequence data. The mean read count was 22,403 ± 12,095 (mean ± standard deviation). The smallest total count of sequences in the sample was 2,299.

### Statistics

Statistical tests were done with R version 4.2.2 using RStudio IDE (36, 37). The quantitative PCR results were compared for both the whole set of samples and the paired samples between the groups (artificial vs natural). The Mann-Whitney U-test was used to compare the qPCR results among all the samples, while the paired *t*-test was used to compare the sample pairs. Co-occurrence network analysis, which calculates the observed and expected frequencies of co-occurrence between each pair of species, was done at the genus level to all samples for both groups, artificial rubber mat and natural rock, using the package cooccur (38). Co-occurrence network analysis gives the number of edges (connections between bacteria) and number of nodes, and each node represents a single genus in the network (39). Indicator species analysis was done on paired samples (A1–A7 and N1–N7) at ASV level using the R package indicspecies.

The Shannon diversity indices and richness were determined for different taxonomic levels (ASV, genus, family, order, class, and phylum) using the vegan (40) and phyloseq R packages (41). For those phyla and classes in which the relative abundance was at least 1%, Shannon indices and richness were determined using function diversity. Data were normalized and transformed to relative abundances by using total sum scaling method, where the individual read counts are divided by the total number of reads and transformed into proportions. To determine the differences between the richness and Shannon indices of all samples, a permutation *t*-test was conducted. For the paired samples, a pairwise permutation *t*-test was used.

The Student’s *t*-test and Mann-Whitney U-test were done to all samples to compare the differences in the bacterial communities between the groups. The Student’s *t*-test was used when the data were normally distributed and the Mann-Whitney U-test when the data were not normally distributed. Depending on the distribution, paired *t*-tests and Wilcoxon signed-rank tests were done to the pairs of rubber mat and natural rock samples. Normality was calculated using the Shapiro-Wilk test (42). If the relative abundance of a bacterial taxon was less than 0.1%, these tests were not done. Differences in the bacterial community composition between the natural rocks and the artificial rubber mats were compared using permutational multivariate analysis of variance (PERMANOVA) (43) with function adonis2 (44) in the vegan package (40) and were visualized with non-metric multidimensional scaling (NMDS). Both were based on the Bray-Curtis dissimilarity. To control the false discovery rate, the Benjamini-Hochberg correction (*Q*-value, significant at the *Q* < 0.05 level) was done to the statistical tests in which multiple comparisons were used (45).

## RESULTS

**Fig 1:**
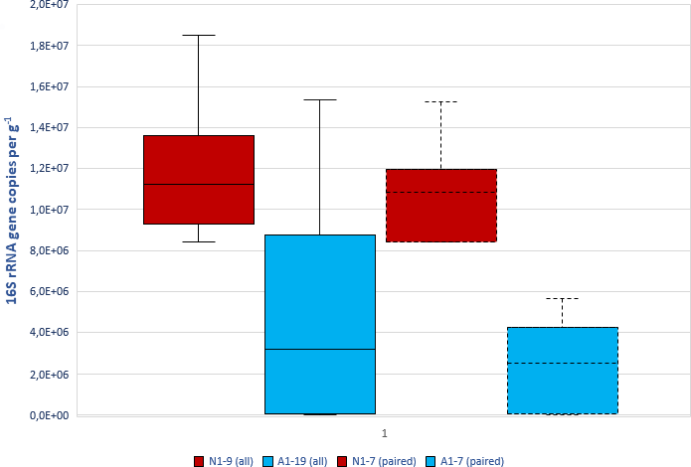
Gene copy numbers in dirt and dust sampled from natural rock (red) and artificial rubber mats (blue) at playgrounds situated in Lahti and Helsinki (Finland). Boxplots with solid outline show 16S rRNA gene copy number in all samples and dotted boxplots in paired samples. Mean (midline), upper and lower hinges (box), and minimum and maximum values (whiskers) are shown.

The 16S rRNA gene copy numbers ranged from 8.43 × 10^6^ to 1.85 × 10^7^ in natural rock samples and from 8.84 × 10^3^ to 1.54 × 10^7^ in artificial rubber mat samples. The average gene copy number in natural rock samples was higher than in rubber mat samples (1.18 × 10^7^ versus 4.63 × 10^6^; U-test *P* = 0.001). The difference between the mean values was even more pronounced in paired samples (1.09 × 10^7^ versus 2.25 × 10^6^; paired *t*-test *P* < 0.0001) (Fig. 1).

**Fig 2:**
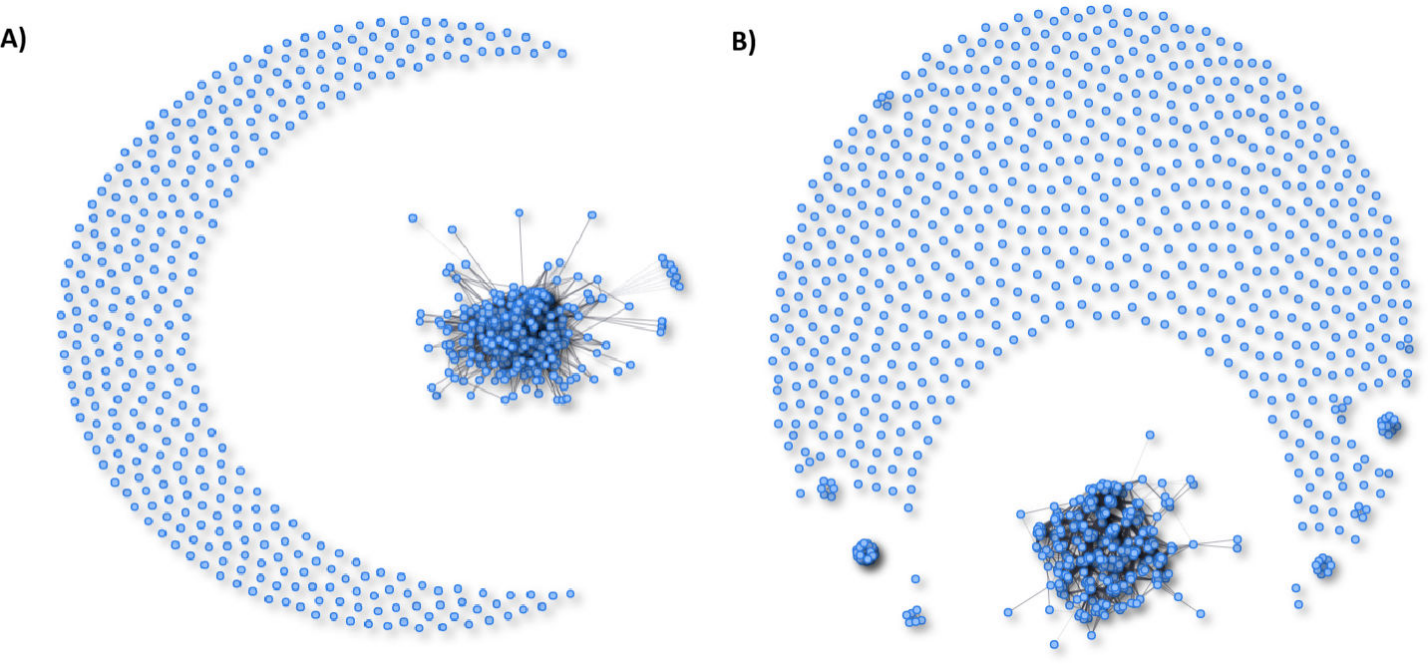
Co-occurrence networks formed by bacteria genera on (A) artificial rubber mats and (B) natural rocks visualized using co-occurrence network analysis. Statistics are shown in Table S2.

Co-occurrence network analyses revealed that bacterial genera had more edges (41,309) on artificial rubber mats than bacterial genera (3,506) on natural rocks. There were also more nodes in the network on artificial rubber mats (644) than on natural rocks (397). In artificial rubber mats, 99.6% of edges were positive and 0.4% were negative. On natural rocks, the same numbers were 95.3% and 4.7%. In the case of artificial rubber mats, bacterial genera formed a single large network, while on natural rocks, bacterial genera formed a large network but also several smaller networks (Fig. 2; Table S2).

Indicator species analysis at the ASV level for paired natural rock and artificial rubber mat samples revealed that the number of species indicative to the rock habitat was much higher (67 ASVs) than the number of species indicative to the rubber mats (3 ASVs) (Table S3). In the rock samples, typical dirt bacteria included taxa from the orders Rhizobiales, Betaproteobacteriales, Sphingobacteriales, and Cytophagales; 26 indicator ASVs belonged to the phylum Proteobacteria and 25 ASVs to the phylum Actinobacteria. There were 8 ASVs belonging to the class Gammaproteobacteria and 14 ASVs belonging to the class Actinobacteria. The three ASVs that were indicative for the artificial rubber mat habitats belonged to the orders Gaiellales, Thermomicrobiales, and Rhizobiales.

**Fig 3:**
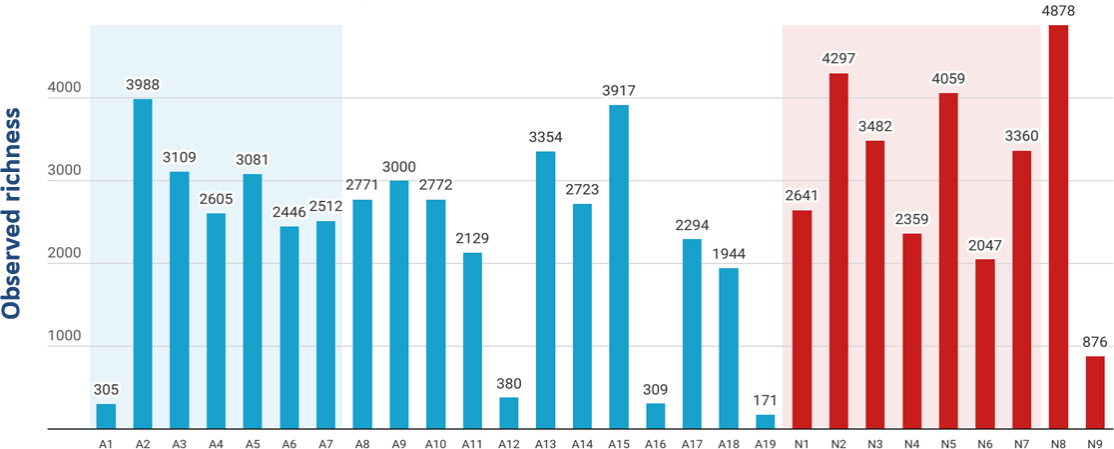
Observed richness of ASVs revealed in dust and dirt sampled from the artificial rubber mats (**A1–A19**) (blue) and natural rocks (**N1–N9**) (red). Paired samples (A1–A7 and N1–N7) are highlighted in the figure.

The average richness (mean ± standard deviation) was higher in natural rock samples (3,111 ± 1,180) compared to the artificial rubber mat samples (2,306 ± 1,189) (Fig. 3; Table S1), which corresponds with results obtained in qPCR analysis. In case of paired samples, the richness was higher in the rock samples (3,178 ± 856) than in rubber mat samples (2,578 ± 1,134).

**Fig 4:**
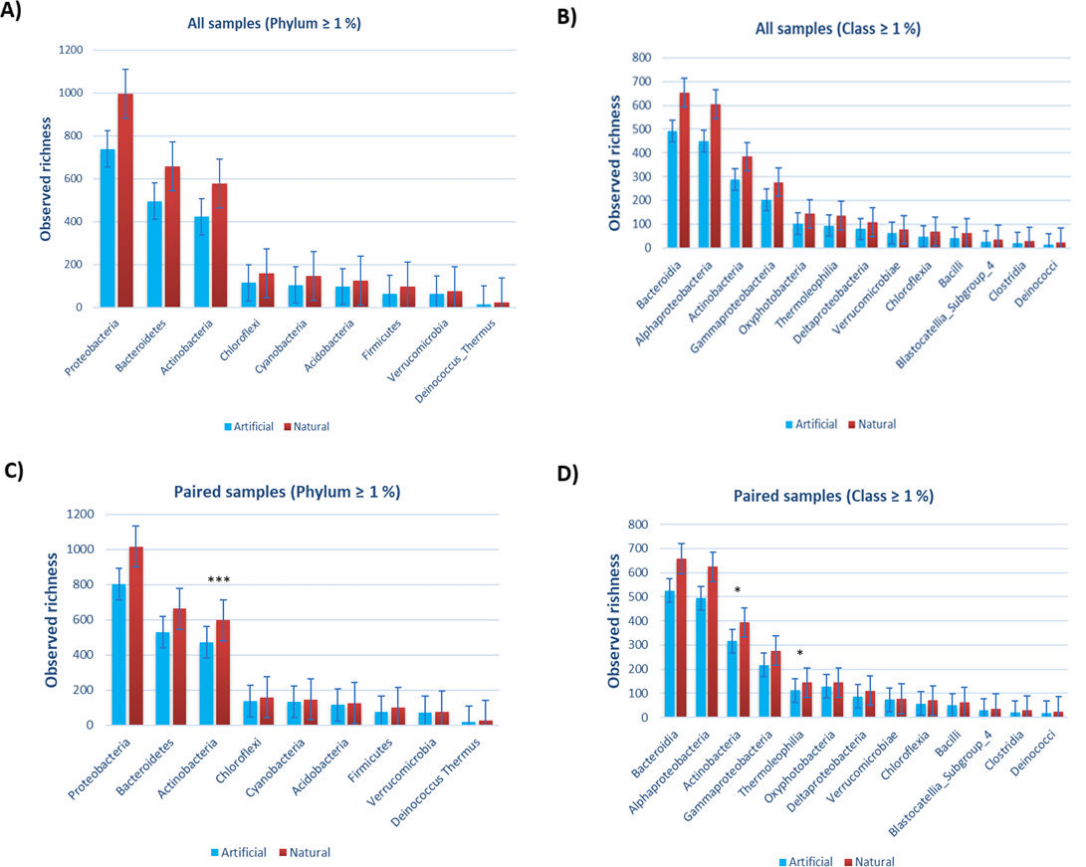
Observed richness of dominating phyla and classes (abundance ≥1%) of bacteria in dust and dirt samples from natural rock- (red) and artificial rubber mat-covered (blue) children’s playgrounds situated in Lahti and Helsinki (Finland). Panels A and B show richness of all samples, and panels C and D show richness of paired samples. Significance codes before *P*-value correction: *P < 0.05, **P < 0.01, and ***P < 0.001. Statistics are shown in Tables S4 and S5.

When the richness of all samples was compared between the groups (natural/artificial) by permutation *t*-test, there were no significant differences in observed richness of the dominant bacterial phyla and classes (relative abundance ≥1%). Instead, when paired samples were compared by pairwise permutation test, the richness of the phylum Actinobacteria was higher in natural rocks than in playground rubber mats (*P* < 0.0001, *Q* < 0.0001). Similarly, the classes Actinobacteria (*P* = 0.016, *Q* = 0.152) and Thermoleophilia (*P* = 0.02, *Q* = 0.152) had a visual difference, although the *Q*-values were not significant. In addition, the visual trend can be seen in all dominating phyla and classes, according to which bacterial communities are richer on natural rocks than on artificial rubber mats (Fig. 4; Tables S4 and S5). The differences were visible when comparing the whole data set as well as when analyzing the paired samples only.

**Fig 5:**
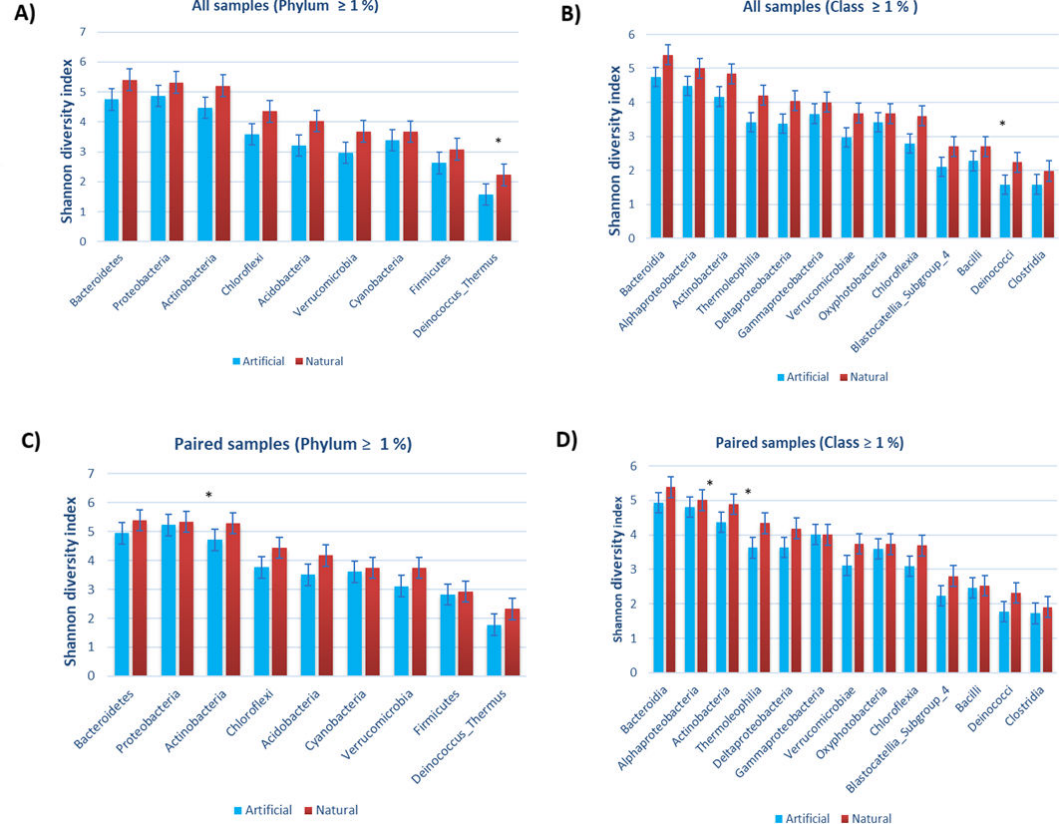
Shannon indices of dominating phyla and classes (abundance ≥1%) of bacteria in dust and dirt samples from natural rock- (red) and artificial rubber mat-covered (blue) children’s playgrounds situated in Lahti and Helsinki (Finland). Panels A and B show Shannon indices of all samples, and panels C and D show paired samples. Significance codes before *P*-value correction: *P < 0.05, **P < 0.01, and ***P < 0.001. Statistics are shown in Tables S7 and S8.

Shannon indices were similar in natural rock and artificial rubber mat samples at different taxonomic levels (phylum, class, genus, and ASV) (Table S6). The indices were also calculated for the paired samples for those phyla and classes which were dominating (relative abundance ≥1%). Interestingly, the results show similar trends as the richness values do; the diversity of the phylum Actinobacteria (*P* = 0.025, *Q* = 0.225) and the classes Actinobacteria (*P* = 0.04, *Q* = 0.406) and Thermoleophilia (*P* = 0.047, *Q* = 0.406) seemed higher on the natural rocks, but no significant differences were left after *P*-value correction, no matter whether all or only paired samples were compared (Tables S7 and S8). Hence, the results are consistent with the richness values. Also, like in bacterial richness, the Shannon diversity indices indicate a trend where diversity of dominant taxa appears to be higher on natural rocks (Fig. 5).

**Fig 6:**
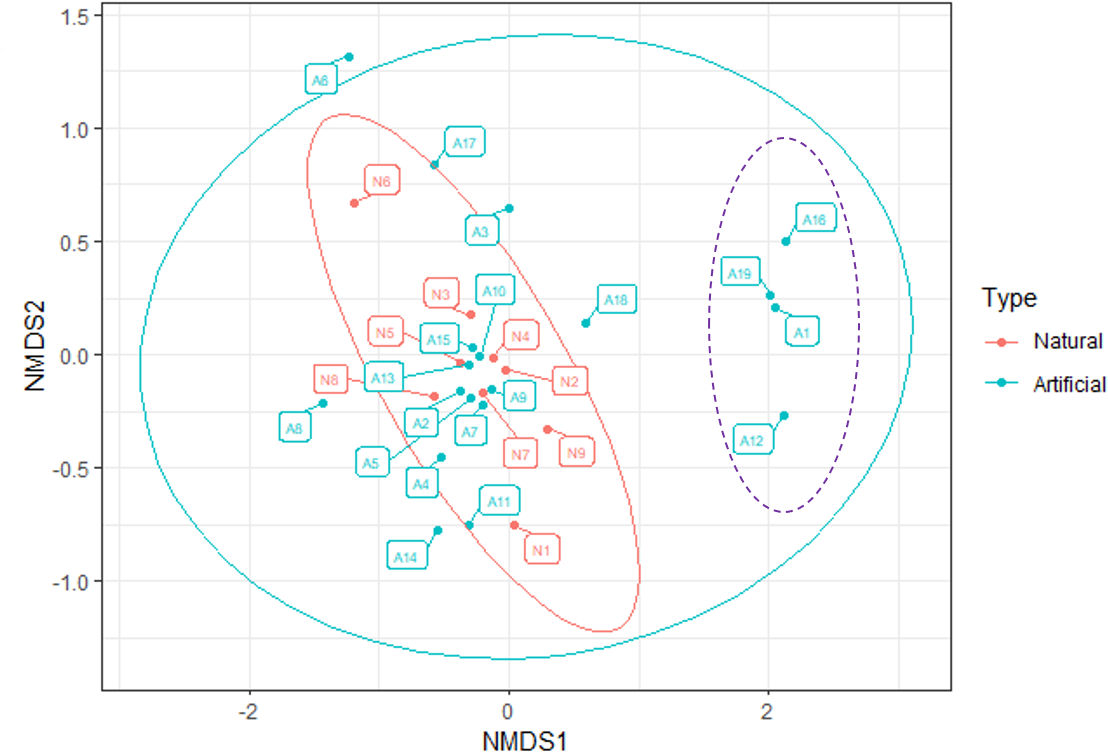
NMDS of the bacterial community composition in the artificial rubber mat samples (blue dots) and the natural rock samples (red dots). Subgroup highlighted with dashed outline (purple). Significance was determined by PERMANOVA (*P* = 0.487, *F* = 0.940, *R*^2^ = 0.035) at the ASV level. Non-metric dimensional scaling ordination based on Bray-Curtis dissimilarity.

According to PERMANOVA (Fig. 6), there were no differences in bacterial community compositions (i.e., beta-diversity) between the artificial rubber mats and natural rocks (*P* = 0.487, *F* = 0.940, *R*^2^ = 0.035). Despite this, natural rock samples’ communities seem to be more similar to each other as compared to communities on artificial rubber mats (Fig. 6). However, no separate groups of natural and artificial samples existed, which indicates no differences between the habitats. Interestingly, four dots presenting samples A1, A12, A16, and A19 (highlighted with purple dashed outline in Fig. 6) form a subgroup. The same samples were characterized by the lowest amount of ASVs (Fig. 3).

## DISCUSSION

The present study focuses on exploring the environmental bacterial communities in children’s play environments within urban areas, framed in the context of planetary health and biodiversity hypothesis. In accordance with our hypotheses, we observed richer and more abundant microbial communities on natural rocks than on playground rubber mats; the naturally dry rock habitats might provide more niches for environmental bacteria compared to artificial rubber mats. Since richness and indicator species analyses revealed poorer Proteobacteria and Actinobacteria composition on rubber mats, the hypothesis that the diversity of bacterial communities is higher on natural rocks was supported. It is worth noting that differences in observed richness of Actinobacteria remained significant only when paired sample tests were used. While the trend was also visible in unpaired samples, it is likely that the variance in the unpaired sample sites is large, which reduces the statistical power.

The assumption that bacterial genera formed different co-occurrence networks on natural rocks versus playground rubber mats was also supported. The observation that the co-occurrence network is more complex on artificial surfaces than on natural rocks is in accordance with the results by Gao et al. (46), who stated that environmental dryness increases bacterial co-occurrences in soil. As environmental conditions become more stressful and challenging, bacteria have been found to exhibit increased complexity and interconnectedness in their networks, as well as a higher propensity to form biofilms (46, 47). The plausible reason is that interactions among microorganisms can serve as a potential mechanism for supplying essential nutrients and creating microscale environmental conditions that allow growth and active metabolism in extremely harsh conditions (46, 48). It has indeed been observed that bacterial species have more interaction with each other and closer integration under more stressful conditions, e.g., alkaline or oligotrophic soils (47). Based on these earlier findings, the results of the current study suggest that playground rubber mats are more stressful habitats for environmental bacteria than the neighboring natural rock tops and plateaus.

We assume that the sources of bacterial abundance in the samples analyzed might be surrounding soil, animals, and plants and bacteria brought on visitors’ shoes, clothes, and hands (49, 50). Especially in the paired samples, these human sources were plausibly similar. The reason for the lower number of bacterial gene copy numbers on the rubber mats might be a lower survival rate on the artificial surfaces as compared with natural ones. This survival rate might be influenced by altered water and air supply as well as materials’ porosity and chemical composition (51–53). Intriguingly, the bacterial gene copy numbers were comparable with those found in soils (54). On the other hand, they were magnitudes lower than the bacterial gene copy numbers found in boreal forest and agricultural soils and at the same level as in commercially available Safety Sand studied by our research group in Finland (4, 55). The current findings may thus support the hypothesis that on playground rubber mats, the bacterial abundance *per se* is too low to facilitate rich and diverse microbial exposure.

Although there was no difference in beta-diversity between the sample types (PERMANOVA) and they did not form separate groups according to the NMDS, the beta-diversity of the samples collected from natural rocks seemed to be less heterogeneous than that of the artificial rubber mats, which had more variation. There was a visually striking, separate subgroup of four samples (A1, A12, A16, and A19). These four samples had the lowest richness values compared to the other samples (Fig. 3). The samples originated from playgrounds on open areas where the distance to the closest woody plants was long. The other rubber mat samples were from playgrounds that had trees next to (approximately 0 m–15 m) the rubber mats. Keeping in mind the subgroup consisted of only four samples, the results hint that green space comprising of woody plants might ameliorate the effect of rubber mats on ground-level microbiota.

The facts that over 95% of ASVs revealed by indicator species analysis were indicative for natural rock samples, and that above 70% of these ASVs belonged to phyla Proteobacteria (26 ASVs) and Actinobacteria (25 ASVs) are interesting from the perspective of health and well-being for several reasons. First, Proteobacteria on human skin have been associated with immunoregulatory functions, proposing a potentially prophylactic effect against allergies and atopic dermatitis, particularly during the first year of life (7, 56). Second, since the low prevalence of Actinobacteria on the skin is connected to skin diseases (57–61), the role of the taxa that are missing in man-made rubber mats may not be insignificant in the context of the biodiversity hypothesis. The view was further enhanced by the fact that at the class level, eight ASVs were classified to the class Gammaproteobacteria, which was earlier distinguished to be associated with increases in plasma TGF-β1 levels and the proportion of regulatory T cells (4, 5, 7).

Three uncultured ASVs which were indicative in artificial rubber mats belonged to Gaiellales, Thermomicrobiales, and Rhizobiales orders. Currently, order Gaiellales is known to contain only a few species, and they are dominant in extreme environments, such as saline-alkaline soils, permafrost sediments, and deep sea habitats (62–64). Similarly, Thermomicrobiales has been identified as a dominant oligotrophic group in saline environments (65). Specifically, the family JG30-KF-CM45 pointed out by our indicator species analysis has been observed in soils where saline irrigation water or saline treatment is present (65, 66). Since winter salting to melt ice at school yards is common in Finland, the found indicator bacteria may be remnants of favorable winter conditions. The third ASV belonged to family Beijerinckiaceae that contains widespread soil methanotrophs and strains fixing atmospheric nitrogen (67). Strains able to biodegrade rubber were previously isolated from the sites containing rubber as solo carbon source together with other compounds and elements essential for microbial growth (68). In this sense, dust and dirt from children’s playgrounds with many rubber microparticles might also be a good hotspot for the selection and development of rubber degraders. To summarize, since the rubber mats microbial community is indicated only by a potential N-fixing methanotroph and two known survivors of N-poor salty environments, it seems that man-made rubber playgrounds lack microscopic niches characteristic of natural dry environments, like rock habitats.

This study also serves as a pilot study to test one of the key assumptions of the biodiversity hypothesis (9). To thoroughly investigate bacterial communities and their diversity on artificial and natural dry surfaces in the context of the biodiversity hypothesis, the samples should be collected in several climate zones and geographic areas, and patterns of shared taxa should be studied (69). This would plausibly ameliorate the limitations related to the sample size of the current study. While searching for school yards in Helsinki and Lahti areas, we realized that the number of natural rocks adjacent to playground rubber mats at school yards limits the number of replicates, and even though there are many rocks favored by locals, often social incoherence or steep slopes prevent children from entering them. These limitations, however, do not lower the value of our findings. On the contrary, it is alarming how easily distinguishable the two bacterial communities are from each other; since playground rubber mats do not contain rich microbiota but merely remnant survivors of neighboring habitats, the results of the current study do encourage reconsidering the widespread use of rubber mats on school yards and other playgrounds. Because microbial richness can benefit human health and well-being through reducing pathogens, remediating soil pollutants, and enhancing immune regulation (4–6, 19, 70–73), preserving and rewilding soil microbial richness should be prioritized in playgrounds and other urban environments.

### Conclusion

The differences found in indicator species and co-occurrence network analyses support the view that playground rubber mats do not support rich microbial communities, even compared to the driest natural habitats found in Finland, natural rocks. In addition, although habitat did not have a major effect on diversity and richness, the differences in dust and dirt microbiome between these two dry habitats were evident and comprised of taxa associated with health in previous studies. Since the current study was not designed to test immune response or commensal microbiota, additional exposure studies are encouraged. Rubber is an essential part of the built environment; it is cheap and relatively safe material, and therefore many playgrounds are built from rubber. Unfortunately, the current study indicates that rubber is far from optimal when it comes to supporting microbial diversity in urban settings. In short, the current study supports the hypothesis that man-made urban environments do not host rich environmental microbiota, which may have an important role from the perspective of planetary health.

### Highlights

- Poor microbiota in the urban environment is assumed to trigger immune-mediated diseases.
- Here, we test if rubber playgrounds host poorer microbiota than neighboring dry rocks.
- Bacterial abundance and richness were higher on natural rocks than rubber mats.
- Species composition and co-occurrence networks indicated high stress on rubber mats.
- The study indicates that modern playgrounds limit exposure to environmental microbiota.

## Data Availability

All bacterial sequence data were deposited into the Sequence Read Archive under BioProject no. PRJNA1037510. All other data needed to support the conclusions of this study are included in the main text and supplemental material.

## SUPPLEMENTAL MATERIAL

Table S1 spectrum.01930-24-s0001.docx Metadata.

Table S2 spectrum.01930-24-s0002.xlsx Co-occurrence network analysis results.

Table S3 spectrum.01930-24-s0003.xlsx Results of indicator species analysis.

Table S4 spectrum.01930-24-s0004.docx Differences of observed richness between paired samples.

Table S5 spectrum.01930-24-s0005.docx Differences of observed richness between all samples.

Table S6 spectrum.01930-24-s0006.docx Shannon diversity indices of all taxonomic levels.

Table S7 spectrum.01930-24-s0007.docx Differences of Shannon indices between all samples.

Table S8 spectrum.01930-24-s0008.docx Differences of Shannon indices between paired samples.

ASM does not own the copyrights to Supplemental Material that may be linked to, or accessed through, an article. The authors have granted ASM a non-exclusive, world-wide license to publish the Supplemental Material files. Please contact the corresponding author directly for reuse.
