# Supplementary material for: Microbial communities on dry natural rocks are richer and less stressed than those on man-made playgrounds
Source: Microbiol Spectr. 2025 Apr 9;13(5):e01930-24. doi: 10.1128/spectrum.01930-24 (PMC12054085; doi:10.1128/spectrum.01930-24)
Supplement: Table S1 — Metadata. [file spectrum.01930-24-s0001.docx]

**Supplement Table 1.** Sample info. Sampling location, city, sample type and sample-specific observed richness are summarized in the table.

| **Sample name** | **Location** | **Type** | **City** | **Richness** |
| --- | --- | --- | --- | --- |
| A1 | Salpakangas school | Artificial | Lahti | 305 |
| A2 | Kiikku playground | Artificial | Helsinki | 3988 |
| A3 | Karisto school track | Artificial | Lahti | 3109 |
| A4 | Karisto school football rink | Artificial | Lahti | 2605 |
| A5 | Ankkurinpuisto playground | Artificial | Lahti | 3081 |
| A6 | Paavola football field | Artificial | Lahti | 2446 |
| A7 | Tiirismaa school basketball court | Artificial | Lahti | 2512 |
| A8 | Lampi playground | Artificial | Helsinki | 2771 |
| A9 | Lotila school 1 | Artificial | Lahti | 3000 |
| A10 | Kärpänen football rink | Artificial | Lahti | 2772 |
| A11 | Kärpänen football goal | Artificial | Lahti | 2129 |
| A12 | Salpakangas school | Artificial | Lahti | 380 |
| A13 | Lotilan school 2 | Artificial | Lahti | 3354 |
| A14 | Lampi playground | Artificial | Helsinki | 2723 |
| A15 | Kiikku playground | Artificial | Helsinki | 3917 |
| A16 | Karisto school, football field | Artificial | Lahti | 309 |
| A17 | Ankkurinpuisto | Artificial | Lahti | 2294 |
| A18 | Karisto school basketball field | Artificial | Lahti | 1944 |
| A19 | Kärpänen school | Artificial | Lahti | 171 |
| N1 | Salpakangas school rock | Natural | Lahti | 2641 |
| N2 | Kiikku rock | Natural | Helsinki | 4297 |
| N3 | Karisto rock 2 | Natural | Lahti | 3482 |
| N4 | Karisto rock 1 | Natural | Lahti | 2359 |
| N5 | Niemi rock 1 | Natural | Lahti | 4059 |
| N6 | Mustankallio rock 2 | Natural | Lahti | 2047 |
| N7 | Mustankallio rock 1 | Natural | Lahti | 3360 |
| N8 | Pirunpesä rock 1 | Natural | Lahti | 4878 |
| N9 | Pirunpesä rock 2 | Natural | Lahti | 876 |
