# Supplementary material for: Microbial communities on dry natural rocks are richer and less stressed than those on man-made playgrounds
Source: Microbiol Spectr. 2025 Apr 9;13(5):e01930-24. doi: 10.1128/spectrum.01930-24 (PMC12054085; doi:10.1128/spectrum.01930-24)
Supplement: Table S4 — Differences of observed richness between paired samples. [file spectrum.01930-24-s0004.docx]

**Supplement Table 4.** Observed richness of dominating phyla and classes (relative abundance ≥ 1 %) in paired samples. Data are presented as mean ± standard deviation. Differences were analyzed with a pairwise permutation t test.

|  | **Artificial** | **Natural** | **P value** | **Q value** |
| --- | --- | --- | --- | --- |
| **Phylum** |  |  |  |  |
| Proteobacteria | 803,7 ± 339,8 | 1016,3 ± 252,7 | 0,07 | 0,316 |
| Bacteroidetes | 530,1 ± 250,1 | 663,4 ± 185,8 | 0,188 | 0,338 |
| Actinobacteria | 473,4 ± 224,8 | 598,1 ± 172,2 | **<0,0001** | **<0,0001** |
| Chloroflexi | 137,1 ± 64,6 | 159,0 ± 48,6 | 0,414 | 0,621 |
| Cyanobacteria | 132,4 ± 61,7 | 147,3 ± 75,1 | 0,602 | 0,773 |
| Acidobacteria | 115,3 ± 69,9 | 125,7 ± 41,8 | 0,766 | 0,805 |
| Verrucomicrobia | 73,4 ± 39,7 | 77,3 ± 28,2 | 0,805 | 0,805 |
| Firmicutes | 74,3 ± 54,9 | 99,4 ± 45,9 | 0,219 | 0,394 |
| Deinococcus_Thermus | 19,6 ± 13,6 | 25,3 ± 6,7 | 0,156 | 0,394 |
|  |  |  |  |  |
| **Class** |  |  |  |  |
| Thermoleophilia | 111,7 ± 60,4 | 145,0 ± 58,2 | **0,023** | 0,152 |
| Actinobacteria | 317,1 ± 144,7 | 393,4 ± 91,9 | **0,016** | 0,152 |
| Alphaproteobacteria | 494,6 ± 209,8 | 624,3 ± 152,0 | 0,07 | 0,279 |
| Bacilli | 50,7 ± 34,0 | 63,3 ± 29,6 | 0,258 | 0,419 |
| Bacteroidia | 526,1 ± 248,7 | 658,6 ± 184,2 | 0,188 | 0,392 |
| Blastocatellia_Subgroup_4 | 29,9 ± 15,8 | 36,3 ± 11,6 | 0,328 | 0,474 |
| Chloroflexia | 58,3 ± 27,0 | 70,3 ± 20,3 | 0,414 | 0,489 |
| Clostridia | 20,0 ± 18,6 | 29,3 ± 17,6 | 0,367 | 0,477 |
| Deinococci | 19,6 ± 13,6 | 25,3 ± 6,7 | 0,156 | 0,392 |
| Deltaproteobacteria | 87,1 ± 45,1 | 110,7 ± 36,3 | 0,086 | 0,279 |
| Gammaproteobacteria | 217,6 ± 104,6 | 276,7 ± 77,0 | 0,211 | 0,392 |
| Oxyphotobacteria | 129,1 ± 60,5 | 144,6 ± 73,2 | 0,539 | 0,584 |
| Verrucomicrobiae | 73,4 ± 39,7 | 77,3 ± 28,2 | 0,805 | 0,805 |
