# Supplementary material for: Microbial communities on dry natural rocks are richer and less stressed than those on man-made playgrounds
Source: Microbiol Spectr. 2025 Apr 9;13(5):e01930-24. doi: 10.1128/spectrum.01930-24 (PMC12054085; doi:10.1128/spectrum.01930-24)
Supplement: Table S5 — Differences of observed richness between all samples. [file spectrum.01930-24-s0005.docx]

**Supplement Table 5.** Observed richness of dominating phyla and classes (relative abundance ≥ 1 %) in all samples. Data are presented as mean ± standard deviation. Differences were analyzed with permutation t test.

| **Phylum** | **Artificial** | **Natural** | **P value** | **Q value** |
| --- | --- | --- | --- | --- |
| Proteobacteria | 737,5 ± 360,3 | 996,4 ± 368,6 | 0,103 | 0,244 |
| Bacteroidetes | 494,6 ± 260,1 | 658,1 ± 279,9 | 0,163 | 0,244 |
| Actinobacteria | 422,1 ± 231,5 | 576,6 ± 213,1 | 0,108 | 0,244 |
| Chloroflexi | 114,9 ± 63,7 | 157,6 ± 75,2 | 0,161 | 0,244 |
| Cyanobacteria | 104,2 ± 61,0 | 146,3 ± 86,2 | 0,208 | 0,267 |
| Acidobacteria | 96,9 ± 66,2 | 125,7 ± 66,9 | 0,298 | 0,336 |
| Verrucomicrobia | 63,6 ± 41,4 | 76,9 ± 39,3 | 0,415 | 0,415 |
| Firmicutes | 64,3 ± 51,4 | 96,2 ± 50,5 | 0,134 | 0,244 |
| Deinococcus_Thermus | 15,6 ± 11,3 | 23,2 ± 8,7 | 0,066 | 0,244 |
|  |  |  |  |  |
| **Class** |  |  |  |  |
| Thermoleophilia | 94,5 ± 63,5 | 135,6 ± 66,9 | 0,143 | 0,241 |
| Actinobacteria | 287,8 ± 146,3 | 384,1 ± 120,3 | 0,088 | 0,241 |
| Alphaproteobacteria | 449,7 ± 216,3 | 605,3 ± 211,0 | 0,090 | 0,241 |
| Bacilli | 41,3 ± 32,3 | 62,3 ± 33,5 | 0,135 | 0,241 |
| Bacteroidia | 491,3 ± 258,4 | 653,2 ± 277,9 | 0,154 | 0,241 |
| Blastocatellia_Subgroup_4 | 26,0 ± 17,6 | 35,4 ± 17,1 | 0,204 | 0,241 |
| Chloroflexia | 49,2 ± 27,1 | 68,3 ± 30,6 | 0,135 | 0,241 |
| Clostridia | 19,8 ± 21,0 | 28,0 ± 17,7 | 0,300 | 0,325 |
| Deinococci | 15,6 ± 11,3 | 23,2 ± 8,7 | 0,064 | 0,241 |
| Deltaproteobacteria | 80,0 ± 47,6 | 109,0 ± 51,8 | 0,175 | 0,241 |
| Gammaproteobacteria | 203,4 ± 109,6 | 277,0 ± 112,0 | 0,128 | 0,241 |
| Oxyphotobacteria | 101,5 ± 60,2 | 143,6 ± 84,3 | 0,193 | 0,241 |
| Verrucomicrobiae | 63,6 ± 41,4 | 76,9 ± 39,3 | 0,422 | 0,422 |
