# Supplementary material for: Microbial communities on dry natural rocks are richer and less stressed than those on man-made playgrounds
Source: Microbiol Spectr. 2025 Apr 9;13(5):e01930-24. doi: 10.1128/spectrum.01930-24 (PMC12054085; doi:10.1128/spectrum.01930-24)
Supplement: Table S6 — Shannon diversity indices of all taxonomic levels. [file spectrum.01930-24-s0006.docx]

**Supplement Table 6.** Shannon diversity indices of all taxonomic levels

| **Taxonomic level** | **Natural** | **Artificial** | **P value** |
| --- | --- | --- | --- |
| **All samples** |  |  |  |
| Phylum | 1.583 | 1.661 | 0.57 |
| Class | 2.206 | 2.079 | 0.47 |
| Genus | 4.809 | 4.257 | 0.09 |
| ASV | 6.251 | 6.167 | 0.89 |
|  |  |  |  |
| **Paired samples** |  |  |  |
| Phylum | 1.717 | 1.747 | 0.836 |
| Class | 2.233 | 2.106 | 0.78 |
| Genus | 4.811 | 4.476 | 0.402 |
| ASV | 6.604 | 6.251 | 0.392 |
