# Supplementary material for: Microbial communities on dry natural rocks are richer and less stressed than those on man-made playgrounds
Source: Microbiol Spectr. 2025 Apr 9;13(5):e01930-24. doi: 10.1128/spectrum.01930-24 (PMC12054085; doi:10.1128/spectrum.01930-24)
Supplement: Table S7 — Differences of Shannon indices between all samples. [file spectrum.01930-24-s0007.docx]

**Supplement Table 7.** Shannon diversity indices of dominating phyla and classes (relative abundance ≥ 1 %) in all samples. Data are presented as mean ± standard deviation. Differences were analyzed with permutation t test.

|  | **Artificial** | **Natural** | **P value** | **Q value** |
| --- | --- | --- | --- | --- |
| **Phylum** |  |  |  |  |
| Proteobacteria | 4,87 ± 1,25 | 5,32 ± 0,46 | 0,233 | 0,300 |
| Bacteroidetes | 4,75 ± 1,12 | 5,41 ± 0,44 | 0,057 | 0,171 |
| Actinobacteria | 4,47 ± 1,31 | 5,21 ± 0,35 | 0,055 | 0,171 |
| Chloroflexi | 3,59 ± 1,36 | 4,35 ± 0,49 | 0,095 | 0,214 |
| Cyanobacteria | 3,39 ± 0,66 | 3,68 ± 0,90 | 0,391 | 0,391 |
| Acidobacteria | 3,21 ± 1,66 | 4,03 ± 0,78 | 0,201 | 0,300 |
| Verrucomicrobia | 2,97 ± 1,52 | 3,68 ± 0,48 | 0,133 | 0,239 |
| Firmicutes | 2,63 ± 1,11 | 3,09 ± 0,94 | 0,270 | 0,304 |
| Deinococcus Thermus | 1,58 ± 0,95 | 2,23 ± 0,41 | **0,028** | 0,171 |
|  |  |  |  |  |
| **Class** |  |  |  |  |
| Thermoleophilia | 3,42 ± 1,28 | 4,21 ± 0,65 | 0,056 | 0,169 |
| Actinobacteria | 4,17 ± 1,17 | 4,84 ± 0,27 | 0,052 | 0,169 |
| Alphaproteobacteria | 4,48 ± 1,22 | 5,00 ± 0,47 | 0,171 | 0,247 |
| Bacilli | 2,27 ± 1,03 | 2,70 ± 0,99 | 0,307 | 0,363 |
| Bacteroidia | 4,75 ± 1,11 | 5,40 ± 0,44 | 0,058 | 0,169 |
| Blastocatellia Subgroup 4 | 2,10 ± 1,18 | 2,71 ± 0,60 | 0,112 | 0,208 |
| Chloroflexia | 2,79 ± 1,42 | 3,60 ± 0,51 | 0,071 | 0,169 |
| Clostridia | 1,58 ± 1,12 | 1,98 ± 0,95 | 0,343 | 0,372 |
| Deinococci | 1,58 ± 0,95 | 2,23 ± 0,41 | **0,027** | 0,169 |
| Deltaproteobacteria | 3,38 ± 1,23 | 4,04 ± 0,48 | 0,078 | 0,169 |
| Gammaproteobacteria | 3,67 ± 1,19 | 4,01 ± 0,42 | 0,299 | 0,363 |
| Oxyphotobacteria | 3,41 ± 0,59 | 3,67 ± 0,90 | 0,444 | 0,444 |
| Verrucomicrobiae | 2,97 ± 1,52 | 3,68 ± 0,48 | 0,133 | 0,216 |
