# Supplementary material for: Microbial communities on dry natural rocks are richer and less stressed than those on man-made playgrounds
Source: Microbiol Spectr. 2025 Apr 9;13(5):e01930-24. doi: 10.1128/spectrum.01930-24 (PMC12054085; doi:10.1128/spectrum.01930-24)
Supplement: Table S8 — Differences of Shannon indices between paired samples. [file spectrum.01930-24-s0008.docx]

**Supplement Table 8.** Shannon diversity indices of dominating phyla and classes (relative abundance ≥ 1 %) in paired samples. Data are presented as mean ± standard deviation. Differences were analyzed with pairwise permutation t test.

|  | **Artificial** | **Natural** | **P value** | **Q value** |
| --- | --- | --- | --- | --- |
| **Phylum** |  |  |  |  |
| Proteobacteria | 5,23 ± 1,07 | 5,34 ± 0,39 | 0,773 | 0,859 |
| Bacteroidetes | 4,95 ± 0,93 | 5,40 ± 0,36 | 0,195 | 0,436 |
| Actinobacteria | 4,71 ± 1,28 | 5,29 ± 0,34 | **0,025** | 0,225 |
| Chloroflexi | 3,77 ± 1,41 | 4,43 ± 0,22 | 0,242 | 0,436 |
| Cyanobacteria | 3,61 ± 0,47 | 3,74 ± 0,85 | 0,641 | 0,824 |
| Acidobacteria | 3,51 ± 1,32 | 4,17 ± 0,49 | 0,109 | 0,436 |
| Verrucomicrobia | 3,12 ± 1,44 | 3,74 ± 0,23 | 0,352 | 0,528 |
| Firmicutes | 2,82 ± 0,92 | 2,93 ± 0,98 | 0,859 | 0,859 |
| Deinococcus_Thermus | 1,78 ± 0,93 | 2,32 ± 0,33 | 0,233 | 0,436 |
|  |  |  |  |  |
| **Class** |  |  |  |  |
| Thermoleophilia | 3,63 ± 1,33 | 4,34 ± 0,46 | **0,047** | 0,406 |
| Actinobacteria | 4,36 ± 1,14 | 4,90 ± 0,27 | **0,040** | 0,406 |
| Alphaproteobacteria | 4,81 ± 1,08 | 5,01 ± 0,33 | 0,539 | 0,757 |
| Bacilli | 2,47 ± 0,92 | 2,53 ± 1,01 | 0,875 | 0,948 |
| Bacteroidia | 4,94 ± 0,93 | 5,39 ± 0,37 | 0,195 | 0,406 |
| Blastocatellia_Subgroup_4 | 2,24 ± 1,13 | 2,81 ± 0,30 | 0,219 | 0,406 |
| Chloroflexia | 3,10 ± 1,39 | 3,69 ± 0,28 | 0,211 | 0,406 |
| Clostridia | 1,72 ± 1,19 | 1,91 ± 1,05 | 0,641 | 0,757 |
| Deinococci | 1,78 ± 0,93 | 2,32 ± 0,33 | 0,156 | 0,406 |
| Deltaproteobacteria | 3,63 ± 0,99 | 4,19 ± 0,27 | 0,125 | 0,406 |
| Gammaproteobacteria | 4,02 ± 0,84 | 4,01 ± 0,34 | 0,984 | 0,984 |
| Oxyphotobacteria | 3,59 ± 0,46 | 3,73 ± 0,85 | 0,625 | 0,757 |
| Verrucomicrobiae | 3,12 ± 1,44 | 3,74 ± 0,23 | 0,352 | 0,571 |
